# Chronic cancer pain in adult oncology centers of Northern Ethiopia: prevalence, determinants, and clinical impact – a multi-center cross-sectional study

**DOI:** 10.1097/MS9.0000000000005373

**Published:** 2026-07-23

**Authors:** Molla A. Tadesse, Eniyew A. Alemu, Haymanot A. Jemeberie, Amare B. Getahun, Henos E. Ashagrie, Temesgen B. Asmare, Yophtahe W. Berhe

**Affiliations:** a Department of Anesthesia, Asrat Woldeyes Health Science Campus, Debre Berhan University, Debre Berhan, Ethiopia; b Department of Anesthesia, University of Gondar, Gondar, Ethiopia; c Department of Optometry, Asrat Woldeyes Health Science Campus, Debre Berhan University, Debre Berhan, Ethiopia; d Department of Anesthesia, College of Medicine and Health Sciences, Debre Tabor University, Debre Tabor, Ethiopia

**Keywords:** cancer, cross-sectional study, chronic pain, Northern Ethiopia, pain management, pain prevalence, pain

## Abstract

**Background:**

Chronic pain significantly affects a person’s quality of life, interferes with treatment adherence, and substantially increases healthcare costs. There are limited data on the prevalence, risk factors, and impacts of chronic pain in Northern Ethiopia.

**Methods:**

Following ethical approval, a multicenter cross-sectional study was conducted in Northern Ethiopia. The impact of chronic cancer pain was assessed using the Brief Pain Inventory Short Form. Binary logistic regression was employed to determine associations with chronic cancer pain. A *P*-value of <0.05 was considered statistically significant.

**Results:**

Among 397 participants, 237 (59.7%) reported chronic cancer pain, and 46.4% reported moderate to severe pain. Chronic cancer pain was significantly associated with female gender [adjusted odds ratio (AOR) = 2.3], primary education (AOR = 2.4), and secondary education (AOR = 2.7), respectively. Breast cancer (AOR = 3.0), gynecologic cancer (AOR = 2.3), lung cancer (AOR = 2.5), genitourinary cancer (AOR = 4.0), and head or neck cancers (AOR = 2.6) also showed positive associations. Additionally, stage IV cancers increased the risk (AOR = 2.2). Patients with chronic cancer pain reported impaired physical activity, increased anxiety and depression, poorer sleep quality, and reduced enjoyment of life compared to those without chronic pain.

**Conclusion:**

Chronic cancer pain is prevalent in Northern Ethiopia, disproportionately affecting women, patients with lower education, and those with specific or advanced cancers. It substantially impairs physical, mental, and overall quality of life. These findings highlight the urgent need for targeted pain management and supportive care strategies to improve outcomes for high-risk cancer patients.

## Introduction

Pain is an unpleasant sensory and emotional experience resulting from actual or potential tissue damage^[^1^]^. The International Association for the Study of Pain defines chronic pain as pain that lasts or recurs for more than three months, encompassing conditions such as cancer-related pain, neuropathic pain, and musculoskeletal pain^[^1^]^. Cancer pain is often caused by tumor expansion that infiltrates or compresses surrounding tissues and activates nociceptors^[^2^]^. In two-thirds to three-quarters of patients, cancer pain is caused by the tumor, while 10–20% of cancer pain is triggered by cancer treatments such as chemotherapy and surgery, and 10% is attributed to comorbidities^[^3,4^]^.

HIGHLIGHTS

- Chronic cancer pain affects approximately 60% of patients with cancer and remains a major public health concern.
- The development of chronic cancer pain is influenced by tumor stage, cancer site, and sociodemographic factors.
- Effective pain assessment and multimodal management strategies are essential for improving patient outcomes and quality of life.

Cancer-related pain is a significant concern and one of the most frequently reported symptoms by cancer patients^[^5^]^. The prevalence of chronic pain in cancer patients ranges from 30.8 to 60%^[^6–8^]^. In the late stages of cancer, 64%^[^9^]^ of patients report chronic pain, which can sometimes reach 75%^[^10^]^ in those with bone metastasis. Among breast cancer patients in South Africa, the prevalence of chronic cancer pain was reported at 59%^[^11^]^.

The Global Burden of Disease Study indicates that pain and pain-related diseases are the leading causes of disability^[^12^]^. Chronic pain negatively affects the quality of life, increases the financial demand on healthcare for both the individual and the health system^[^13^]^, and contributes to heightened anxiety, anger, and feelings of depression, thereby further diminishing a patient’s quality of life^[^9–12^]^. Factors including young age^[^14^]^, axillary node dissection for breast cancer treatment^[^15^]^, metastatic cancer^[^16^]^, chemotherapy^[^17^]^, surgery^[^18^]^, and medical comorbidities^[^19^]^ increase the risk of chronic cancer pain. Additionally, low education levels and low socioeconomic status^[^20^]^ are associated with a higher risk of chronic cancer pain.

Existing studies have several limitations, indicating the need for further research. A study was conducted in South Africa focusing on breast cancer patients^[^11^]^, which limits its applicability to other types and locations of cancer. The small sample size and single-center setting of the study further restrict the generalizability of its findings. Moreover, the study exclusively involved female participants, overlooking potential gender-specific variations in the experience and management of chronic cancer pain. This gap emphasizes the necessity for local data in resource-limited settings. Therefore, the aim of this study was to determine the prevalence, associated factors, and impacts of chronic cancer pain among adult patients at four oncologic centers in Northern Ethiopia.

## Methods

### Study design, area, period, and population

A multicenter cross-sectional study was conducted at four hospitals in Northern Ethiopia that can provide oncologic services: University of Gondar Comprehensive Specialized Hospital, Dessie Comprehensive Specialized Hospital (BB), Tibebe Ghion Comprehensive Specialized Hospital, and Felege Hiwot Comprehensive Specialized Hospital. These hospitals serve the northern region of the country, including Amhara, Afar, and Tigray. Participants were recruited from the chemotherapy clinics of these facilities between March and May 2023. The study was conducted in compliance with the Strengthening the Reporting of Cohort, Cross-Sectional, and Case-Control Studies in Surgery (STROCSS) guideline ^[^21^]^.

University of Gondar Comprehensive Specialized Hospital has an adult oncology ward with a bed capacity of 28, serving approximately 450 patients per month with the assistance of three oncologists and 12 oncology nurses. Furthermore, Dessie Comprehensive Specialized Hospitalserves 350 patients monthly within its 10-bed oncology unit. The oncology service is provided by one oncologist, three oncology nurses, and three general nurses. Meanwhile, Tibebe Ghion Comprehensive Specialized Hospital serves about 300 patients monthly, with treatment delivered by three oncologists and four oncology nurses. Finally, Felege Hiwot Comprehensive Specialized Hospital offers services with 22 beds for oncology patients, attending to about 325 patients monthly, with two oncologists, two oncology nurses, and seven general nurses providing oncology care.

The source population for this study consisted of all patients with a histologic diagnosis of cancer at the four cancer treatment centers. Adult patients aged 18 years and older with a histologic diagnosis of cancer were included in the study. In contrast, uncooperative patients, those with a diagnosis of psychiatric illness, and patients admitted with a diagnosis of oncologic emergencies were excluded from the study.

### Outcome measures and operational definitions

The primary outcome of the study was the prevalence of chronic cancer pain. Chronic cancer pain is defined as pain lasting more 3 months that is caused by the primary cancer, metastatic cancers, or post-cancer treatments, including surgery, chemotherapy, radiotherapy, or a combination of these^[^22^]^. Cancer-related pain was defined as pain associated with the cancer diagnosis itself, medical tests such as biopsies, or treatments like surgery and chemotherapy side effects^[^23^]^.

Chronic cancer pain is defined as pain lasting more than 3 months that is caused by primary cancer itself, metastatic cancers, and post-cancer treatments, including surgery, chemotherapy, radiotherapy, or a combination of these ^[^24^]^. The Numeric Rating Scale (NRS) was used to assess the severity of pain in study participants^[^25^]^. Participants scored their pain on an 11-point scale ranging from 0 (“no pain”) to 10 (“worst pain”). Pain severity was graded as 0 (“no pain”), 1–3 (“mild pain”), 4–6 (“moderate pain”), and 7–10 (“severe pain”)^[^26^]^. Since the severity of chronic pain changes throughout the day and night, participants were asked to rate their current, least, worst, and average pain scores over the past 24 hours using the NRS^[^26^]^. The worst pain score, which showed the best correlation with functional interference, was used to diagnose chronic cancer pain^[^27^]^. In this study, chronic cancer pain was defined as a worst self-reported pain severity score of 1 or greater on the NRS in the past 24 hours that persisted or recurred for more than 3 months. For this particular study, participants who met these criteria were considered to have chronic cancer pain.

Additionally, the secondary outcomes of the study included identifying independent variables for chronic cancer pain and determining the physical and emotional impact of chronic cancer pain.

The first group of independent variables that affect chronic cancer pain consists of sociodemographic factors, including age, gender, level of education, residency, and marital status. Furthermore, other variables include behavioral factors such as alcohol consumption and cigarette smoking. For this study, alcohol drinking was defined as individuals who consumed 12 or more alcoholic beverages, including any combination of cans of beer, glasses of wine, or mixed drinks^[^28^]^. The second group of factors includes clinical variables such as comorbidities, tumor stage, metastasis, and tumor site. The last group of independent variables comprises treatment regimens for cancer (chemotherapy, radiotherapy, surgery, or combinations thereof) and the type of prescribed analgesic agent.

Participants were also assessed for the impact of pain on general activity, walking ability, normal work, mood (anxiety, depression), enjoyment of life, sleep, and relationships with others using the Brief Pain Inventory Short Form^[^29^]^. Interference was classified as 0 (“no”), 1–3 (“mild”), 4–6 (“moderate”), and 7–10 (“severe”)^[^26^]^. Sleep disturbances were defined as difficulty falling asleep (taking more than 30 minutes to fall asleep) or difficulty staying asleep (experiencing more than 30 minutes of nocturnal waking)^[^30^]^.

### Sample size determination and sampling technique

We determined the sample size for this binary logistic regression analysis using the formula described by Vittinghoff *et al*^[^31^]^. We considered the proportion of chronic cancer pain to be 50%, the multiple correlation to be 0.19, the marginal probability of the outcome to be 0.7, and the margin of error to be 0.05. The calculated sample size was 384. After adding a 5% non-response rate, the final sample size increased to 404. We employed proportional allocation and consecutively selected 128 participants from University of Gondar Comprehensive Specialized Hospital, 92 from Dessie Comprehensive Specialized Hospital, 85 from Tibebe Ghion Comprehensive Specialized Hospital, and 99 from Felege Hiwot Comprehensive Specialized Hospital.

### Ethical approval, data collection, processing, and analysis

The ethical review committee of the School of Medicine at the University of Gondar Comprehensive Specialized Hospital provided ethical approval (reference number: UoG/SM/06/01/4097/2015). As the research involved human subjects, it adhered to the principles outlined in the Declaration of Helsinki^[^32^]^. We developed the study questionnaire by adapting items from previous research^[^7,11,26,33^]^. Data were collected through structured interviews and a review of medical records during outpatient and inpatient visits to the chemotherapy clinics of each hospital. One trained data collector at each cancer treatment center handled the data collection. Written informed consent was obtained from all participants who were able to read and write, while fingerprint signatures were secured from those who could not. The study was conducted with adult participants capable of providing consent; as a result, legal guardians were not involved.

The principal investigator reviewed the data for completeness, clarity, and accuracy. Data entry was performed using EpiData software version 4.6 to ensure accuracy and consistency, and statistical analysis was conducted using STATA version 17. Data cleaning addressed inconsistencies and improved accuracy, while data coding reduced complexity and enhanced comparability. Chronic cancer pain was coded into a yes/no option using the patient-reported NRS pain scores. The age-standardized prevalence of chronic cancer pain was computed using the World Health Organization (WHO) 2000–2025 standard population^[^34^]^. Participants’ ages were categorized based on previous studies to ensure comparability^[^35^]^. The Shapiro–Wilk test was used to assess data distribution, the variance inflation factor evaluated multicollinearity, and the Hosmer–Lemeshow test checked model fit. Additionally, we examined the group difference between patients with and without chronic cancer pain in health-related quality of life using the Mann–Whitney *U* test. Chi-square tests or Fisher’s exact test and binary logistic regression were performed to examine associations between dependent and independent variables. Independent variables with a *P*-value below 0.2 in bivariable binary logistic regression were included in the multivariable analysis. In the final analysis, variables with a *P*-value of less than 0.05 were deemed statistically significant. The strength of the association between chronic pain and independent variables was assessed using adjusted odds ratios (AOR) with a 95% confidence interval (CI).

Utilization of artificial intelligence

We do not use artificial intelligence for either research or manuscript writing.

## Result

### Sociodemographic characteristics of study participants

In this study, 397 participants were included, resulting in a response rate of 98.3%. The median age of the participants was 48 years, with an interquartile range of [38, 59] years. The age distribution revealed that the largest proportion of individuals with chronic cancer pain were between 39 and 59 years old, 100 (42.2%). A large proportion of study participants reporting chronic cancer pain were female, 146 (61.6%), and most participants reporting chronic cancer pain were married, 180 (75.9%). Additionally, 125 (52.7%) of participants with chronic *cancer* pain lived in rural areas, and 87 (36.7%) with chronic cancer pain had not received formal education. Lastly, 227 (95.8%) and 206 (86.9%) of study participants who experienced chronic cancer pain had no history of excessive alcohol consumption and had no history of cigarette smoking, respectively (Table 1).

**Table 1:** Sociodemographic characteristics of study participants by chronic cancer pain, n = 397.

Characteristics	Chronic cancer pain *n* (%)		*P*-value
	Yes, *n* = 237 (59.7)	No, *n* = 160 (40.3)	
Age			0.069
18–38 years	75 (31.6)	38 (23.8)	
39–59 years	100 (42.2)	86 (53.7)	
60–84 years	62 (26.2)	36 (22.5)	
Gender			0.013
Female	146 (61.6)	78 (48.7)	
Male	91 (38.4)	82 (51.3)	
Marital status			0.436
Married	180 (75.9)	125 (78.1)	
Single	22 (9.3)	17 (10.6)	
Divorced	23 (9.7)	15 (9.4)	
Widowed	12 (5.1)	3 (1.9)	
Residency			1.000
Urban	112 (47.3)	75 (46.9)	
Rural	125 (52.7)	85 (53.1)	
Level of education			0.047
Did not attend formal education	87 (36.7)	56 (35)	
Attend primary education	76 (32.1)	49 (30.6)	
Attend secondary education	53 (22.4)	26 (16.2)	
Attend college and above education	21 (8.8)	26 (16.2)	
Alcohol consumption			0.261
Yes	10 (4.2)	11 (6.9)	
No	227 (95.8)	149 (93.1)	
Smoking			0.043
Yes	31 (13.1)	10 (6.3)	
No	206 (86.9)	150 (93.7)	

### Clinical characteristics of study participants

The findings indicate that most study participants with chronic cancer pain, 197 (83.1%), have no systemic diseases. Regarding the type of cancer, 67 (28.3%) of participants with chronic cancer pain were diagnosed with breast cancer. In terms of cancer stage, 91 (38.4%) of patients who reported chronic cancer pain had stage II cancers. Furthermore, 230 (97%) of patients with chronic pain were diagnosed with metastasized cancer. Additionally, chemotherapy was the most common treatment regimen for 169 (71.3%) of study participants experiencing chronic cancer pain. Lastly, non-opioids were the prescribed analgesics for 84 (35.4%) of patients with chronic cancer pain (Table 2).

**Table 2:** Clinical characteristics of study participants by chronic cancer pain, n = 397.

Characteristics	Chronic cancer pain *n* (%)		
	Yes, *n* = 237 (59.7)	No, *n* = 160 (40.3)	*P*-value
Systemic illness			0.073
Yes	40 (16.9)	39 (24.4)	
No	197 (83.1)	121 (75.6)	
Site of cancer			0.001
Breast	67 (28.3)	25 (15.6)	
Gynecologic	48 (20.3)	22 (13.8)	
Lung	17 (7.2)	9 (5.6)	
Hematologic	12 (5.1)	17 (10.6)	
Head and neck	13 (5.5)	7 (4.4)	
Genitourinary	16 (6.8)	6 (3.8)	
Other cancers	11 (4.6)	13 (8.1)	
Gastrointestinal	61 (25.7)	53 (33.1)	
Cancer stage			
Stage IV	54 (22.8)	23 (14.4)	0.046
Stage III	58 (24.5)	50 (31.3)	
Stage II	91 (38.4)	56 (35)	
Stage I	34 (14.3)	31 (19.4)	
Metastasis			
Yes	230 (97)	15 (9.4)	0.007
No	7 (3)	145 (90.6)	
Treatment regimen for cancer			0.030
Chemotherapy	169 (71.3)	123 (76.9)	
Surgery and chemotherapy	63 (26.6)	28 (17.5)	
Other treatments	5 (2.1)	9 (5.6)	
Type of analgesia			0.001
Weak opioids	70 (29.5)	44 (27.5)	
Weak opioid with non-opioids	60 (25.3)	72 (45)	
Strong opioid with non-opioids	23 (9.7)	11 (6.9)	
Non-opioids	84 (35.4)	33 (20.6)	

Other cancers: central nervous system, skin, thymus gland, otolaryngology, pregnancy-related, orthopedic, and cancer of unknown primary origin. Other treatments:^[^3^]^ surgery^[^2^]^, radiotherapy^[^1^]^, chemotherapy and radiotherapy^[^4^]^, chemotherapy, surgery and radiotherapy^[^2^]^ symptomatic and/or chemotherapy^[^5^]^.

### Prevalence of chronic cancer pain

The crude prevalence of chronic cancer pain was 237 (59.7%, 95% CI: 54.8–64.4). The study showed the variation in the prevalence of pain across age groups: 75 (31.6%) for participants aged 18–38 years, 100 (42.2%) for those aged 39–59 years, and 62 (26.2%) for those aged 60–84 years. The age-standardized prevalence of chronic cancer pain was approximately 237 (59.6%, 95% CI: 54.9–64.5). The largest proportion of study participants, 127 (53.6%), reported mild pain; 91 (38.4%) reported moderate pain; and 19 (8%) reported severe pain.

### Comparison of health-related quality of life between individuals with and without chronic pain

The findings of the Mann–Whitney *U* test revealed significant differences in health-related quality of life between participants with and without chronic cancer pain. Participants experiencing chronic cancer pain reported significant (*P* < 0.01) difficulties in general activity, walking ability, normal work, anxiety, depression, and sleep compared to those without chronic pain (*P* < 0.001). However, there was no significant difference in relationships with other individuals (*P* = 0.730) (Table 3).

**Table 3:** Comparison of health-related quality of life between individuals with and without chronic pain, *n* = 397.

Health related quality of life	Chronic cancer pain			*P*-value
	Yes	No	*U* statistic	
General activity			8237.5	< 0.001
No problems	24 (10.1)	85 (53.1)		
Mild problems	139 (58.7)	73 (45.6)		
Moderate problems	66 (27.8)	2 (1.3)		
Severe problems	8 (3.4)	0		
Walking ability			10 387	< 0.001
No problems	11 (4.6)	62 (38.7)		
Mild problems	88 (37.1)	54 (33.8)		
Moderate problems	86 (36.3)	32 (20)		
Severe problems	52 (21.9)	12 (7.5)		
Normal work			30 723.5	< 0.001
No problems	7 (3)	75 (46.9)		
Mild problems	78 (32.9)	61(38.1)		
Moderate problems	98 (41.3)	12 (7.5)		
Sever problems	54 (22.8)	12 (7.5)		
Anxiety			8871.5	< 0.001
None	10(4.2)	65 (40.6)		
Mild	26 (11)	30 (18.8)		
Moderate	145 (61.2)	57 (35.6)		
Severe	56 (23.6)	8 (5)		
Depression			29 121.5	< 0.001
No	4 (1.7)	77 (48.1)		
Mild	126 (53.2)	58 (36.3)		
Moderate	95 (40.1)	19 (11.9)		
Severe	12 (5)	6 (3.8)		
Enjoyment of life			12 952.5	< 0.001
None	21 (9)	46 (28.7)		
Mild	79 (33.3)	55 (34.4)		
Moderate	89 (37.5)	50 (31.3)		
Severe	48 (20.2)	9 (5.6)		
Sleep			18 751	< 0.01
None	74 (31.2)	53 (33.1)		
Mild	137 (57.8)	88 (55)		
Moderate	18 (7.6)	18 (11.3)		
Severe	8 (3.4)	1 (0.6)		
Relation with other people			18,595	0.7304
None	58 (24.5)	37 (23.1)		
Mild	94 (39.7)	72 (45)		
Moderate	80 (33.8)	47 (29.4)		
Severe	5 (2.1)	4 (2.5)		

### Factors associated with chronic cancer pain

In bivariable logistic regression analysis, gender, level of education, type of cancer, and stage of cancer met the threshold for inclusion in the multivariable binary logistic regression analysis (*P* < 0.2). In the final model, gender, level of education, type of cancer, and stage of cancer were statistically associated with chronic cancer pain (*P* < 0.2). The odds of chronic cancer pain in female patients were higher compared to male patients (AOR = 2.3, CI: 1.2–2.1, *P* = 0.043). Attending primary education (AOR = 2.4, CI: 1.1–4.1, *P* = 0.041) and secondary education (AOR = 2.7, CI: 1.2–5.9, *P* = 0.013) were associated with a higher risk of chronic cancer pain compared to patients with a college education or higher. Additionally, compared to patients with gastrointestinal cancer, those with breast (AOR = 3.0, CI: 1.6–5.9, *P* = 0.001), gynecologic (AOR = 2.3, CI: 1.1–4.9, *P* = 0.020), lung (AOR = 2.5, CI: 1.1–6.3, *P* = 0.042), head or neck (AOR = 2.6, CI: 1.3–7.2, *P* = 0.046), and genitourinary (AOR = 4.0, CI: 1.4–11.8, *P* = 0.009) cancers had a higher risk of chronic cancer pain. Lastly, stage IV cancers increased the risk of chronic cancer pain (AOR = 2.2, CI: 1.1–4.7, *P* = 0.032) compared to patients diagnosed with stage I cancers (Table 4).

**Table 4:** Factors associated with chronic cancer pain, n = 397.

Characteristics	COR, 95% CI	Crude *P*-value	AOR, 95% CI	Adjusted *P*-value
Gender				
Female	1.7(1.1–2.5)	0.012	2.3 (1.2–2.1)	0.043
Male	1.00		1	1
Level of education				
Did not attend formal education	1.8 (1.0– 3.5)	0.051	1.8 (0.9–3.7)	0.088
Attend primary education	1.9(1.0–3.8)	0.049	2.4 (1.1–4.1)	0.041
Attend secondary education	2.5(1.2–5.3)	0.015	2.7 (1.2–5.9)	0.013
Attend college and above	1		1	1
Site of cancer				
Breast	3.1 (1.7–5.6)	0.000	3 (1.6–5.9)	0.001
Gynecologic	2.5 (1.3–4.7)	0.004	2.3 (1.1–4.9)	0.020
Lung	2.2 (1.9–5.3)	0.086	2.5 (1.1–6.3)	0.042
Hematologic	0.8 (0.4–1.9)	0.622	0.8 (0.4–2.0)	0.704
Head and neck	2.1 (1.8–5.8)	0.04	2.6 (1.2–7.2)	0.046
Genitourinary	3 (1.1–8.4)	0.029	4 (1.4–11.8)	0.009
Other cancers	1 (0.4–2.4)	0.953	1 (0.4–2.2)	0.780
Gastrointestinal	1		1	1
Cancer stages				
Stage IV	2.1 (1.1–4.3)	0.030	2.2 (1.1–4.7)	0.032
Stage III	1.1 (1.2–1.7)	0.859	1.0 (0.5–1.9)	0.916
Stage II	1.5 (0.8–2.7)	0.191	1.7 (0.9–3.4)	0.090
Stage I	1		1	1

AOR, adjusted odds ratio; CI, confidence interval; COR, Crud odds ratio.

## Discussion

The objective of this study is to determine the prevalence, associated factors, and impacts of chronic cancer pain on physical activities, emotions, and sleep disturbances among adult patients in Northern Ethiopia. In this study, the age-standardized prevalence of chronic cancer pain was 59.6% (95% CI: 54.9–64.5). This finding is in line with a similar study conducted among breast cancer patients^[^11^]^. The inclusion of various cancer types in our study, which have different risks for developing chronic pain, may explain the stabilization of the pain score. However, the prevalence of chronic cancer pain in this study is lower than that reported in other previous research, where chronic cancer pain prevalence ranged from 75 to 90%^[^10,36^]^. This discrepancy may be attributed to those studies including a higher number of participants with advanced cancer stages and participants diagnosed with head or neck cancers, which are associated with chronic cancer pain due to the rich nerve supply in the head and facial areas, compared to the current study, which included various cancer types.

In the current study, gender, level of education, cancer site, and cancer stage were statistically associated with chronic cancer pain. Recent research in acute pain management has indicated potential gender differences in pain sensitivity, tolerance, and the effectiveness of analgesics, with some chronic pain conditions occurring primarily in females^[^37^]^. Our findings reveal that participants with only primary and secondary education have a higher risk of developing chronic cancer pain compared to those who attended college or university. This result is consistent with other studies^[^38,39^]^. This trend may be attributed to better treatment adherence^[^40,41^]^, increased knowledge about cancer pain and analgesics^[^42^]^ and a more positive attitude toward cancer pain management^[^38,40^]^ among individuals with higher education levels. Additionally, another study found that educational interventions reduced pain levels and addressed patient-related barriers to effective pain management^[^43^]^.

Regarding the site of cancer and chronic pain, this study demonstrated that the highest prevalence of chronic pain was reported in patients with breast cancer, gynecologic cancers, and gastrointestinal cancer. Additionally, the study showed a significant association between chronic cancer pain and breast cancers, gynecologic cancers, head or neck cancers, genitourinary cancers, and lung cancers. These findings are supported by a similar study conducted after maxillectomy for cancer treatment^[^36^]^. The association between head and neck cancer and chronic pain can be attributed to the rich innervation of the orofacial region, which facilitates various bodily functions that are constant triggers of pain^[^44^]^. The association between breast cancer and chronic cancer pain is supported by existing evidence^[^39^]^. Similarly, a single-center study reported that the highest prevalence of chronic cancer pain was found in patients with gastrointestinal cancer^[^45^]^. The causes of chronic pain in cancer are associated with the type of tumor, the invasiveness of the tumor, the time since cancer treatment, and the cancer treatment regimen^[^46^]^. In the present study, the high prevalence of chronic cancer pain in stage II cancers may be due to a higher proportion of stage II cancer participants compared to those in other cancer stages. Additionally, there is a statistically significant association between stage IV cancer and chronic pain, a finding supported by another study^[^47^]^.

Chronic cancer pain severely compromises the quality of life for patients and their families, affects treatment adherence, and reduces survival rates^[^48^]^. This study demonstrated that a large proportion of participants experienced impaired physical activities, as measured by general activity, walking ability, and normal work. These findings are supported by similar studies that report cancer pain interferes with daily functioning^[^49–51^]^. In the current study, approximately half of the participants with chronic cancer pain reported moderate to severe anxiety, while 45% reported moderate to severe depression. The findings of this study are supported by a previous study conducted on patients with peripheral neuropathy^[^52^]^. Depression and anxiety can also worsen pain^[^53,54^]^. Moreover, studies have revealed that pain levels are significantly higher in depressed cancer patients^[^55,56^]^. The mechanism behind chronic pain-induced depression is attributed to decreased levels of monoamines, including serotonin, dopamine, and norepinephrine^[^57^]^ On the other hand, anxiety and depression contribute to the development of chronic pain by increasing pain catastrophizing^[^58^]^. This study also revealed that participants with chronic pain had statistically significant differences in trouble sleeping and enjoyment of life. Studies have shown that there is a bidirectional interaction between sleep and pain. Pain and sleep disturbances accounted for a significant proportion of the variance in quality of life (*P* < 0.001), with the highest proportion observed in predicting global health status (*P* < 0.001)^[^59^]^. On the other hand, disturbed or short sleep duration are factors that increase sensitivity to pain stimuli (hyperalgesia)^[^60^]^.

### Strengths and limitations of the study

This study has several potential strengths. The study generates local data in the context of northwest Ethiopia, addressing multiple components, including the prevalence and risk factors of chronic cancer pain, as well as its physical and emotional impacts, such as physical activity, anxiety, depression, and sleep disturbances. The finding of the Mann–Whitney *U* test, which showed a statistically significant association between chronic cancer pain and its physical and emotional impacts, further supports the results obtained from the binary logistic regression analysis. However, the study has limitations. As a cross-sectional study, it does not establish causality between chronic cancer pain and the associated factors or its physical and emotional impacts. Since we utilized self-reported questionnaires, the study’s results may be influenced by recall biases and subjective interpretations.

## Conclusion

The study highlights that the majority of participants (59.7%) experienced chronic cancer pain. Factors such as female gender, lower levels of education, specific cancer types (breast, gynecologic, lung, and head or neck cancers), and stage IV cancers were statistically associated with the development of chronic cancer pain. Additionally, chronic pain and its physical and emotional impacts were found to have a statistically significant positive association.

## Recommendation

To reduce the high prevalence of chronic cancer pain and improve patients’ quality of life, we recommend targeted educational interventions for vulnerable groups, particularly individuals with lower levels of education. Additionally, it is recommended to provide psychological support to reduce participants’ anxiety and depression, as well as to improve sleep quality related to pain. Specialized pain management strategies and palliative care services should be provided for patients with high-risk cancer types prone to the development of chronic cancer pain and for patients with advanced-stage cancers. To ensure timely intervention, routine physical and emotional assessments must be conducted. Policy efforts should focus on improving access to pain management resources and medications.

## Data Availability

The corresponding author can provide the data and materials used in this study upon request.

## Research registration unique identifying number (UIN)

This research is registered with a unique identifying number of research registry 11670.
